# Using artificial intelligence to predict sexual health outcomes in endometriosis: a decision tree model algorithm

**DOI:** 10.1093/sexmed/qfag013

**Published:** 2026-03-23

**Authors:** Alexandre Vallée, Anis Feki, Jean-Marc Ayoubi

**Affiliations:** Department of Epidemiology and Public Health, Foch Hospital, Suresnes 92150, France; Department of Gynecology and Obstetrics, University Hospital of Fribourg, 1708 Fribourg, Switzerland; Department of Obstetrics, Gynecology and Reproductive Medicine, Foch Hospital, Suresnes 92150 France; Medical School, University of Versailles, Saint-Quentin-en-Yvelines (UVSQ), 78180 Versailles, France

**Keywords:** endometriosis, FSFI, sexual dysfunction, decision tree model, artificial intelligence, machine learning, pain, dyspareunia, infertility, BMI

## Abstract

**Background:**

Endometriosis profoundly impairs sexual function through complex interactions between pain, hormonal disturbances, psychological distress, and sociodemographic factors.

**Aim:**

To develop and validate a decision tree–based model identifying key predictors of sexual dysfunction in women with endometriosis.

**Methods:**

We conducted a cross-sectional online survey among 1586 women with endometriosis recruited through social media in France between November 2023 and January 2024. Participants completed sociodemographic and clinical questionnaires and the Female Sexual Function Index (FSFI), with sexual dysfunction defined as FSFI < 26.55. The predictors included pain characteristics, menstrual symptoms, digestive symptoms, infertility, BMI, lifestyle factors, and treatment history. A classification and regression tree model was trained on 70% of the sample and validated on the remaining 30%. Model performance was assessed using area under the receiver operating characteristic curve (AUC), sensitivity, and specificity. Subgroups at high or low risk of sexual dysfunction were identified through terminal nodes (rules) of the decision tree.

**Outcomes:**

The primary outcome was classification into sexual dysfunction versus normal sexual function.

**Results:**

Of the 1586 respondents, 1358 (86%) met the criteria for sexual dysfunction. The decision tree demonstrated strong discrimination in the training dataset (AUC = 0.96; sensitivity = 98%; specificity = 82%) and acceptable performance in the validation dataset (AUC = 0.79; sensitivity = 72%; specificity = 77%). Chronic pelvic pain, dyspareunia, worsening pain over time, heavy menstrual bleeding, infertility, BMI, digestive symptoms, education level, and treatment history were identified as major determinants. Several terminal nodes showed a 100% probability of sexual dysfunction, most notably women with severe menstrual cramps, heavy menstrual bleeding, no treatment, normal weight or obesity, infertility, or pronounced digestive symptoms. Conversely, women with no dyspareunia, no urinary or bowel pain, high education, and overweight exhibited a 0% probability of dysfunction.

**Clinical Implications:**

AI-driven decision trees can support early identification of high-risk profiles and guide individualized management strategies to improve sexual health in women with endometriosis.

**Strengths and Limitations:**

Strengths include a large sample, comprehensive symptom profiling, and transparent model interpretability. Limitations include reliance on self-reported diagnosis, potential selection bias inherent to online recruitment, lack of geographic and clinical verification data, and the cross-sectional nature of the analysis, preventing causal inference.

**Conclusion:**

A decision tree–based model accurately identified key predictors of sexual dysfunction in endometriosis, supporting its potential for personalized risk stratification and clinical decision support.

## Introduction

Endometriosis is defined as a disease characterized by the presence of endometrium-like epithelium and/or stroma outside the endometrium and myometrium, usually associated with an inflammatory process.^1–3^ It is considered a chronic inflammatory condition, defined as the presence of endometrium-like tissue outside the uterus.^4^ The establishment and growth of such ectopic tissue are estrogen-dependent,^5^ explaining why the disease predominantly affects women of reproductive age. However, its clinical consequences and management may persist well into the postmenopausal period.^6^ The inflammatory and estrogen-dependent nature of endometriosis contributes to progressive lesion development, central sensitization, and persistent pain, thereby explaining the chronic and often progressive course of the disease.^7^^,^^8^

Common symptoms include intense menstrual pain (dysmenorrhea), pain during intercourse (dyspareunia), abdominal and pelvic discomfort that may extend to the lower back, as well as pain during urination and gynecological assessments.^9–11^

The exact prevalence of endometriosis is unknown but estimates range from 2% to 10% in the general female population and up to 50% among infertile women.^12^^,^^13^ Thus, it is estimated that currently at least 190 million women and adolescent girls worldwide are affected by the disease during reproductive age, although some women may suffer beyond menopause.^14^^,^^15^ While not all women with endometriosis are symptomatic, endometriosis-associated pain and infertility are the clinical hallmarks of the disease affecting not only women with endometriosis, but also their partners and families. An impact of endometriosis, and particularly pain symptoms, has been shown on quality of life, but also on a range of activities and life domains, including physical functioning, everyday activities and social life, education and work, sex, intimacy and intimate partnerships, and mental health and emotional well-being.^16^ Moreover, it is diagnosed in nearly half of the patients experiencing chronic pelvic pain syndrome.^17^ However, because some individuals remain asymptomatic, the true prevalence is difficult to determine, posing challenges to precise epidemiological evaluation.^18^^,^^19^

In assessing sexual function, the Female Sexual Functioning Index (FSFI), a comprehensive 19-item questionnaire where higher scores denote better sexual function, has been employed.^20^ According to findings by Shi et al., women diagnosed with endometriosis exhibited significantly lower FSFI scores in comparison to their healthy counterparts, indicating impaired sexual well-being.^21^ Beyond physical symptoms, endometriosis can impact various dimensions of a woman’s life, including sexual relationships, often resulting in increased relational stress.^22^ Evidence suggests that the symptoms of endometriosis can disrupt intimate and sexual partnerships.^23^^,^^24^ The aspect of sexuality, which encompasses both psychosocial and physiological dimensions, plays a crucial role in not only a woman’s physical health but also her psychological state and interpersonal connections.

With the emergence of personalized, predictive, and preventive medicine, there is an increasing need to identify the determinants that negatively affect QoL in individuals with endometriosis. Gaining deeper insight into these factors can facilitate the development of targeted interventions aimed at enhancing patient outcomes and overall well-being. Advances in artificial intelligence (AI), particularly through decision tree models, present promising opportunities for refining analyses related to endometriosis.^25–28^ However, to date, no decision tree models have been employed to explore sexual function among women with endometriosis. Consequently, the objective of this study was to construct a decision tree–based model to assess sexual function by incorporating various factors associated with endometriosis.

## Methods

### Study design and participants

We designed and conducted a cross-sectional survey using survey software developed by our hospital.^29^ The survey was completed anonymously to encourage honest and unbiased responses.

The study link was disseminated via social media (Instagram), where participants were asked to forward this link to others they know. All the registrants were free to accept or decline the invitation, with no monetary reward received in return. Participants were also informed that they could withdraw at any time. Following internationally accepted ethical codes, respondents were duly informed of the purpose of the survey and were reminded of their participation rights before proceeding to take the survey. A research protocol was conducted to obtain approval from an ethical committee. The distribution of the questionnaire occurred between November 2023 and January 2024 in France on social media (Instagram). We closed the survey link after the workshop ended.

### Questionnaire and measuring instruments

The questionnaire was divided into the following sections:

Sociodemographic questions (couple status, age, educational level, children).

Body mass index (BMI) was calculated as weight (kg)/height^2^ (m^2^) and initially classified according to WHO criteria as underweight (<18.5 kg/m^2^), normal weight (18.5-24.9 kg/m^2^), overweight (25.0-29.9 kg/m^2^), and obesity (≥30 kg/m^2^). For descriptive analyses and decision tree modeling, and due to the number of participants, categories were grouped into normal BMI (<25 kg/m^2^, including underweight and normal weight; only 89 participants showed BMI < 18.5 kg/m^2^), overweight BMI (25.0-29.9 kg/m^2^), and obesity BMI (≥30 kg/m^2^). Obesity subclasses (class I-III) were not analyzed separately due to limited sample sizes in extreme categories.

Questions related to the disease (diagnosis, symptoms, treatment, age of diagnosis, etc.)

Symptoms of endometriosis were defined as follows: pain during sexual intercourse (dyspareunia); heavy menstrual bleeding; infertility, defined as a disease characterized by the failure to establish a clinical pregnancy after 12 months of regular and unprotected sexual intercourse^30^; pain during urination (dysuria), particularly during menstruation; pain during bowel movements, particularly during menstruation (dyschezia); other digestive symptoms such as diarrhea, constipation, or nausea; worsening pain over time; excessive menstrual cramps or chronic pelvic pain; and medical and surgical treatment for endometriosis.

Heavy menstrual bleeding was defined as menstrual bleeding lasting more than 7 days, requiring frequent change of sanitary protection (eg, soaking one or more pads or tampons per hour for several consecutive hours), nighttime changes, or the presence of large blood clots, in accordance with American College of Obstetricians and Gynecologists / National Institute for Health and Care Excellence (ACOG/NICE) criteria.

Female Sexual Function Index questionnaire: The FSFI contains 19 items and collects data on 6 domains of sexual function: desire, arousal, vaginal lubrication, orgasm, satisfaction, and pain. For each domain except the pain domain, the item scores range from 0 to 5. Higher item scores indicate better function. Items in the pain domain are coded by a descending scale. To obtain the total FSFI score, the item scores within each domain are added and then multiplied by a correction factor. The resulting scores within each of the 6 domains are added to obtain a total FSFI score. Higher scores reflect better sexual function.^20^ An FSFI total score under 26.55 was considered to be the optimal cut score for differentiating women with sexual dysfunction from women without sexual dysfunction.^31^

### Statistical analysis

 Characteristics of the study population were described using mean ± standard deviation (SD) for continuous variables. Categorical variables were described as numbers and proportions. Comparisons between groups were performed using the Mann-Whitney test or Student *t*-test for continuous variables. Pearson’s *χ*^2^ test was performed for categorical variables. The statistical analyses were performed using SAS software (version 9.4; SAS Institute, Carry, NC). A *P*-value < 0.05 was considered statistically significant.

### Decision tree model

The target or outcome variable consisted of two classes: one class for the sexual dysfunction and the second for good sexual functioning. Data mining detects unknown patterns or prediction rules. One of the different methods of data mining is the decision tree. The decision tree model is a nonparametric methodology that performs tree-based classification modeling.^32^^,^^33^ The main purpose of this methodology is to provide a predictive tool for the target variable regardless of the predictors. Decision tree models are composed of three types of nodes: root node, internal node, and end node.^34^ This methodology performs splitting criteria to break a node to form a tree. The internal variables of the model represent a tree structure in which a decision is performed on each branch according to the data features. Splitting criteria provide a rate for each predictive variable. Variables that have the best rate of splitting criteria are selected as staying in the algorithm. In the decision tree, the first variable or root node is the main important determinant, and then the other variables could be classified in order of importance.^32^ The root node is the variable that can divide the whole population with the highest information gain.

The classification and regression tree (CART) is a decision tree algorithm.^35^ CART is made by splitting subsets of data using all the predictor variables. By this procedure, all the root nodes are created repeatedly. The CART algorithm creates a binary division of the tree and prunes the tree on the cost complexity. The CART algorithm uses the Gini impurity index to select the best variable.

The Gini impurity measures the probability of misclassifying a randomly chosen element. It is defined as:


$$ G=1-\sum_{i=1}^C{p}_i^2 $$

where $C$ is the number of classes and ${p}_i$ is the proportion of samples in the node that belong to class $i$.

For a split into left ($L$) and right ($R$) child nodes, the weighted Gini impurity is:


$$ {G}_{\mathrm{split}}=\frac{N_{\mathrm{L}}}{N}{G}_{\mathrm{L}}+\frac{N_{\mathrm{R}}}{N}{G}_{\mathrm{R}} $$

where ${G}_{\mathrm{L}}$ and ${G}_{\mathrm{R}}$ are the Gini impurities of the left and right child nodes; ${N}_{\mathrm{L}}$ and ${N}_{\mathrm{R}}$ are the number of samples in the left and right child nodes, respectively; and$N$ is the total number of samples in the parent node. The best split is chosen by minimizing the weighted Gini index.

Gini gain:


$$ \Delta G={G}_{\mathrm{parent}}-{G}_{\mathrm{split}} $$

where ${G}_{\mathrm{parent}}$ is the Gini impurity before splitting and ${G}_{\mathrm{split}}$is the weighted impurity after splitting. A larger $\Delta G$ indicates a better split.

All the variables were entered in the decision tree model regardless their significance.

Due to the small number of participants with classes of obesity, obesity subclasses were not modeled separately in the decision tree analysis to preserve model stability and avoid unreliable terminal nodes.

The model was trained on a testing population (70% of the overall population) and validated on a validation population (30% of the overall population).

## Results

A total of 1586 women with endometriosis responded to the online questionnaire, and among them, 759 (48%) have children, 1247 (79%) were in couples, and 410 (26%) declared smoking tobacco. Their overall mean FSFI level was 17 (SD: 9) and BMI was 26 (SD: 6). When considering the dichotomization of our study population according to good sexual function or not, we observed significant differences for age (*P* = .042), being in a couple, (*P* < .001), and pain during urination during periods (*P* < .001) (Table 1).

**Table 1:** Characteristics of the study population according to sexual function status.

**Variable**	**Sexual dysfunction (N = 1358) n/mean**	**%/SD**	**No sexual dysfunction (N = 228) n/mean**	**%/SD**	***P*-value**
**Age (years)**					.042
18-25	209	15.39%	28	12.28%	
26-30	266	19.59%	56	24.56%	
31-35	279	20.54%	48	21.05%	
36-40	289	21.28%	46	20.18%	
41-45	204	15.02%	22	9.65%	
>45	111	8.17%	28	12.28%	
**Surgical treatment of endometriosis**	204	15.02%	35	15.35%	.898
**Education level**					.172
High	331	24.41%	56	24.56%	
Moderate	246	18.14%	53	23.25%	
Low	779	57.45%	119	52.19%	
**Children**	641	47.41%	118	51.75%	.225
**In a couple**	1037	76.36%	210	92.11%	<.001
**Current smoking**	351	25.92%	59	26.34%	.896
**Heavy menstrual bleeding**	871	64.14%	141	61.84%	.506
**Infertility**	433	31.89%	65	28.51%	.306
**Pain during urination during menstruation**	688	50.66%	88	38.60%	<.001
**Pain during bowel movements during menstruation**	905	66.64%	139	60.96%	.097
**Other digestive symptoms (diarrhea, constipation, nausea)**	1202	88.51%	203	89.04%	.817
**Worsening pain over time**	990	72.90%	166	72.81%	.976
**Severe menstrual cramps**	877	64.58%	142	62.28%	.504
**BMI category**					.965
Obesity (≥30 kg/m^2^)	312	23.11%	52	22.81%	
Class III (≥40 kg/m^2^)	21	1.56%	2	0.88%	
Class II (35-39.9 kg/m^2^)	88	6.52%	12	5.26%	
Class I (30-34.9 kg/m^2^)	203	15.04%	38	16.67%	
Overweight (25-29.9 kg/m^2^)	346	25.63%	57	25.00%	
Normal weight (<25 kg/m^2^)	692	51.26%	119	52.19%	
**BMI (kg/m** ^**2**^ **)**	26.0	5.79	25.6	5.62	.287
**Medical treatment for endometriosis**	589	43.50%	90	39.47%	.254
**FSFI total score**	14.7	8.41	28.5	1.32	<.001
FSFI domains					
Desire	4.25	2.01	7.50	1.56	<.001
Arousal	6.78	5.56	17.1	2.07	<.001
Vaginal lubrication	7.71	6.37	17.5	2.55	<.001
Orgasm	5.46	4.63	13.4	1.68	<.001
Satisfaction	6.85	4.66	12.0	1.49	<.001
Pain	7.22	4.54	8.67	1.72	<.001

**Figure 1:**
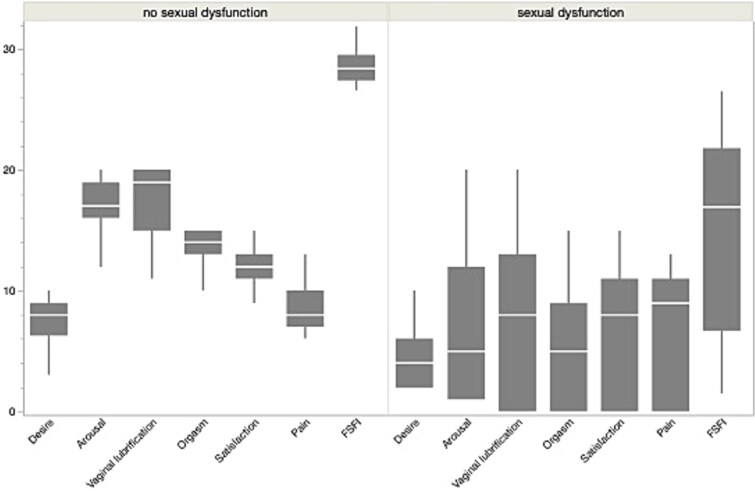
Distribution of Female Sexual Function Index (FSFI) total score and domain scores in women with and without sexual dysfunction (FSFI < 26.55 [no sexual dysfunction] vs ≥26.55 [sexual dysfunction]).

Figure 1 shows the different items of the FSFI score according to the two groups.

**Figure 2:**
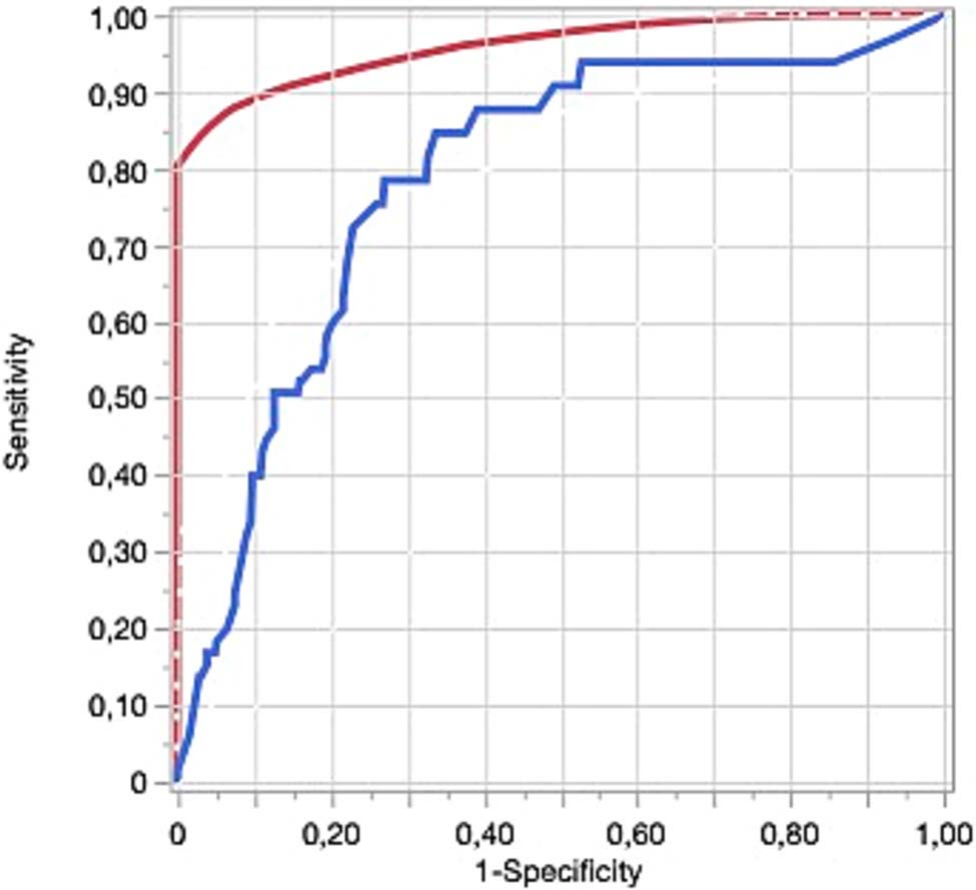
Area under the receiver operating characteristic curve (ROC AUC) of the decision tree model for the testing phase (red line, AUC = 0.96) and for the validation phase (blue line, AUC = 0.79).

A decision tree model was performed on the testing population (70% of the overall population, area under the receiver operating characteristic curve [AUC] = 0.96, sensitivity = 98%, and specificity = 82%) and validated on the validation population (30% of the overall population, AUC = 0.79, sensitivity = 72%, and specificity = 77%) (Figure 2).

The decision tree model identified 23 main subgroups of women who consistently exhibited either marked deterioration or no impairment in sexual functioning, based on their clinical and sociodemographic profiles (Table 2).

**Table 2:** Rules created by the decision tree model, final number of participants by rule with the probability of sexual dysfunction associated with each rule and then, the different steps of each rule.

**Rules**	**Final number**	**Probability of sexual dysfunction (%)**	**All steps of the rule**
**Rule 1**	**N = 5**	**0**	If, in couple, no pain during urination during periods, age < 46 years, pain, particularly excessive menstrual cramps felt, no heavy menstrual bleeding, other digestive issues (diarrhea, constipation, nausea), no treatment of endometriosis, high education
**Rule 2**	**N = 8**	**88**	If, in couple, no pain during urination during periods, age < 46 years, pain, particularly excessive menstrual cramps felt, no heavy menstrual bleeding, other digestive issues (diarrhea, constipation, nausea), treatment against endometriosis, pain during bowel movements during periods
**Rule 3**	**N = 7**	**100**	If, in couple, no pain during urination during periods, age < 46 years, pain, particularly excessive menstrual cramps felt, no heavy menstrual bleeding, no other digestive issues (diarrhea, constipation, nausea)
**Rule 4**	**N = 5**	**20**	If, in couple, no pain during urination during periods, age < 36 years, no pain, particularly excessive menstrual cramps felt, no heavy menstrual bleeding, no other digestive issues (diarrhea, constipation, nausea), no tobacco smoking, pain during bowel movements during periods
**Rule 5**	**N = 5**	**0**	If, in couple, no pain during urination during periods, age > 46 years, no pain, particularly excessive menstrual cramps felt, no heavy menstrual bleeding, no other digestive issues (diarrhea, constipation, nausea), age > 46 years, worsening pain over time
**Rule 6**	**N = 5**	**0**	If, in couple, no pain during urination during periods, age < 46 years, pain, particularly excessive menstrual cramps felt, heavy menstrual bleeding, no infertility, obesity or overweight, low education, treatment against endometriosis, having children
**Rule 7**	**N = 8**	**12**	If, in couple, no pain during urination during periods, age < 36 years, no pain, particularly excessive menstrual cramps felt, heavy menstrual bleeding, no pain during bowel movements during periods, other digestive issues (diarrhea, constipation, nausea)
**Rule 8**	**N = 5**	**100**	If, in couple, no pain during urination during periods, age < 46 years, pain, particularly excessive menstrual cramps felt, heavy menstrual bleeding, no infertility, overweight, low education, no treatment against endometriosis
**Rule 9**	**N = 16**	**100**	If, in couple, no pain during urination during periods, age < 46 years, pain, particularly excessive menstrual cramps felt, heavy menstrual bleeding, no infertility, normal weight, moderate or low education, no treatment against endometriosis
**Rule 10**	**N = 18**	**100**	If, in couple, no pain during urination during periods, age < 46 years, pain, particularly excessive menstrual cramps felt, heavy menstrual bleeding, infertility, obesity or overweight
**Rule 11**	**N = 7**	**16**	If, in couple, no pain during urination during periods, tobacco smoking, treatment against endometriosis, age < 40 years, not having children, high education
**Rule 12**	**N = 12**	**100**	If, in couple, no pain during urination during periods, no pain, particularly excessive menstrual cramps felt, no heavy menstrual bleeding, other digestive issues (diarrhea, constipation, nausea), high or moderate education, no tobacco smoking, no pain during bowel movements during periods, age > 36 years
**Rule 13**	**N = 41**	**100**	If, in couple, no pain during urination during periods, no pain, particularly excessive menstrual cramps felt, no heavy menstrual bleeding, other digestive issues (diarrhea, constipation, nausea), low education
**Rule 14**	**N = 12**	**100**	If, in couple, no pain during urination during periods, age > 40 years, surgical treatment of endometriosis, overweight or normal weight
**Rule 15**	**N = 5**	**0**	If, in couple, no pain during urination during periods, tobacco smoking, pain during bowel movements during periods, treatment against endometriosis, age < 40 years, having children
**Rule 16**	**N = 11**	**88**	If, in couple, no pain during urination during periods, tobacco smoking, pain during bowel movements during periods, treatment against endometriosis, age < 40 years, not having children, high education
**Rule 17**	**N = 8**	**0**	If, in couple, pain during urination during periods, no tobacco smoking, other digestive issues (diarrhea, constipation, nausea), no surgical treatment of endometriosis, age < 40 years, worsening pain over time, low education, no infertility, heavy menstrual bleeding, having children
**Rule 18**	**N = 16**	**100**	If, in couple, pain during urination during periods, tobacco smoking, pain during bowel movements during periods, no treatment against endometriosis, worsening pain over time, having children, obesity or overweight
**Rule 19**	**N = 9**	**100**	If, in couple, pain during urination during periods, no tobacco smoking, age < 40 years, other digestive issues (diarrhea, constipation, nausea), no surgical treatment of endometriosis, worsening pain over time, infertility
**Rule 20**	**N = 21**	**100**	If, in couple, pain during urination during periods, no tobacco smoking, age < 40 years, other digestive issues (diarrhea, constipation, nausea), no surgical treatment of endometriosis, worsening pain over time, high education, no treatment against endometriosis, heavy menstrual bleeding, not having children
**Rule 21**	**N = 16**	**100**	If, not in couple, age < 36 years, other digestive issues (diarrhea, constipation, nausea), worsening pain over time, no tobacco smoking, treatment against endometriosis, overweight or normal weight, heavy menstrual bleeding
**Rule 22**	**N = 25**	**100**	If, not in couple, age < 36 years, other digestive issues (diarrhea, constipation, nausea), worsening pain over time, no tobacco smoking, no treatment against endometriosis, moderate or low education
**Rule 23**	**N = 33**	**100**	If, not in couple, age < 36 years, other digestive issues (diarrhea, constipation, nausea), worsening pain over time, tobacco smoking

Women with painful menstrual cramps, heavy menstrual bleeding, and no infertility, combined with normal weight, moderate/low education, and no treatment for endometriosis, exhibited 100% sexual dysfunction (rules 9 and 10). Those with pain during urination and bowel movements, worsening pain over time, and obesity also showed 100% probability of sexual dysfunction (rule 18). Women who underwent surgical treatment of endometriosis, had normal weight or were overweight, and were over 40 years old had a 100% probability of sexual dysfunction (rule 14). Patients with digestive issues (diarrhea, constipation, nausea), worsening pain over time, and tobacco use showed 100% sexual dysfunction, especially if they were not in a couple and under 36 years old (rules 21, 22, and 23). Women in a couple who had digestive symptoms, no surgical treatment of endometriosis, high education, and worsening pain over time also had 100% probability of sexual dysfunction (rule 20).

It should be noted that terminal nodes with extreme probabilities (eg, 100%) reflect specific multivariable combinations of predictors identified by the CART algorithm and do not imply causal effects of any single variable, including surgical treatment. In particular, surgical history appeared in different terminal nodes depending on the surrounding clinical context (age, pain severity, digestive symptoms, education level), highlighting interaction effects rather than isolated associations.

Women who were in a couple, had no pain during urination or bowel movements, no severe menstrual cramps, and high education showed 0% probability of sexual dysfunction (rules 1 and 5). Those with no worsening pain over time, not overweight, no heavy menstrual bleeding, and who had received endometriosis treatment also had 0% sexual dysfunction (rule 6). Being in a couple appeared to be a protective factor, as women not in a couple and experiencing digestive symptoms and worsening pain over time had 100% sexual dysfunction (rules 21 and 22). Women who were in a couple, had pain during bowel movements, but were younger than 40 and had high education showed an 88% probability of sexual dysfunction (rule 16). Women with menstrual cramps, heavy menstrual bleeding, overweight, and low education had an 88% probability of sexual dysfunction (rule 8) (Table 2).

## Discussion

### Interest of AI application

The use of decision tree models to examine factors affecting sexual function in women with endometriosis offers a novel and valuable approach to addressing this complex issue. Traditional statistical analyses often fall short in capturing the multifactorial nature of sexual dysfunction, which involves interactions between clinical symptoms, psychological distress, and sociodemographic influences. In contrast, decision trees provide a transparent, data-driven method that identifies the most relevant predictors and organizes them in a structured hierarchy.^36^ By systematically analyzing these variables, decision trees can detect intricate relationships that may not be evident through conventional methods. This ability to model nonlinear and interdependent factors makes them particularly well suited for evaluating sexual dysfunction in endometriosis, a condition known for its heterogeneous presentation. Additionally, decision pathways generated by AI models offer clinicians practical insights that can improve the individualization of therapeutic approaches.^37^^,^^38^ The integration of machine learning into sexual health assessments could significantly enhance the ability to predict, prevent, and manage sexual dysfunction in affected patients.^39^

A major strength of decision tree modeling is its capacity to uncover complex interactions between diverse factors such as dyspareunia, infertility, BMI, emotional distress, and relationship dynamics. Unlike traditional linear models, which assume predictor independence, decision trees can identify nuanced subgroups of patients with varying degrees of sexual dysfunction. For instance, the model may highlight an unexpected link between overweight, severe menstrual cramps, and psychological distress, where increased awareness of symptoms contributes to greater sexual impairment.^40^ These findings illustrate how AI can reveal previously unrecognized risk factors, paving the way for more effective interventions.

From a clinical perspective, leveraging decision tree models allows for a targeted and stratified approach to managing sexual dysfunction in endometriosis patients. By ranking risk factors according to their influence on sexual health, healthcare providers can prioritize interventions for women at the highest risk. For example, if the model identifies severe pelvic pain and infertility as leading contributors, personalized strategies such as pelvic floor rehabilitation, hormonal management, and psychosexual therapy can be recommended. Additionally, the visual representation of patient profiles created by decision trees simplifies shared decision-making,^41^ ensuring that both patients and clinicians can better understand individualized treatment options. Moreover, incorporating AI-driven models into digital health platforms could enable real-time patient monitoring, enhancing the precision and adaptability of treatment plans.

Ultimately, integrating AI-based decision tree models into endometriosis research provides a powerful tool for decoding the complexities of sexual dysfunction. By utilizing advanced data-driven methodologies, researchers and clinicians can gain deeper insights into the underlying mechanisms, enhance the early detection of high-risk groups, and implement tailored therapeutic strategies. This approach holds great potential to improve the overall management of sexual health in women with endometriosis, offering more effective, individualized, and proactive interventions.

### Determinants of sexual dysfunction in women with endometriosis

Endometriosis is a complex gynecological condition with a profound impact on multiple aspects of quality of life, particularly sexual function. The decision tree model in this study provided a data-driven approach to identifying key clinical, psychological, and sociodemographic predictors of sexual dysfunction in women with endometriosis. The results revealed that pain progression,^42^ menstrual disorders,^43^ infertility,^44^ digestive symptoms,^45^ and treatment history were critical determinants influencing sexual well-being.

### Pain as a central determinant of sexual dysfunction

Chronic pelvic pain, particularly worsening pain over time and pain during urination or bowel movements, emerged as a strong predictor of impaired sexual function.^46^ This finding aligns with previous research suggesting that persistent pain leads to avoidance behaviors, fear of intercourse (anticipatory pain anxiety), and reduced sexual desire.^47^ The neuropathic nature of endometriosis-related pain can cause central sensitization, leading to heightened pain perception and further impairing sexual activity.^48^

Furthermore, pain during intercourse (dyspareunia), a hallmark symptom of deep infiltrating endometriosis, can lead to psychosexual distress, reduced arousal, and relationship strain.^49^ Women with dyspareunia and worsening pain over time had a high probability of sexual dysfunction, emphasizing the need for early pain management strategies through multimodal approaches, including pharmacological, surgical, and psychological interventions.^50^^,^^51^

### Menstrual disorders and hormonal influence on sexual function

Heavy menstrual bleeding was another key factor contributing to sexual dysfunction.^52^ In this study, women experiencing heavy menstrual bleeding and painful cramps were significantly more likely to report worse FSFI scores. Excessive menstrual bleeding may lead to fatigue, anemia, and reduced libido, while severe menstrual cramps (dysmenorrhea) further exacerbate pain-related sexual avoidance.^53^

From a hormonal perspective, estrogen dominance in endometriosis is known to increase inflammation, pain severity, and menstrual abnormalities, further impacting sexual health.^54^ Hormonal treatments, including progestins, gonadotropin-releasing hormone (GnRH) analogues, and combined oral contraceptives, play a critical role in reducing symptoms and improving sexual function.^55^ However, some treatments, such as GnRH analogues, may have adverse effects on libido due to estrogen suppression, highlighting the importance of personalized treatment strategies.^56^

### Infertility as a psychological burden affecting sexual well-being

Infertility is a well-documented source of psychological distress in women with endometriosis^57^ and was identified as a major predictor of poor sexual function in this study. Women with infertility and obesity or overweight had a high probability of sexual dysfunction, underscoring the emotional toll associated with reproductive concerns.^58^

The bidirectional relationship between infertility and sexual dysfunction is well established.^59^^,^^60^ Women facing infertility often experience decreased sexual spontaneity, increased performance pressure, and emotional distress, all of which negatively affect sexual desire and satisfaction.^61^^,^^62^ Psychological interventions, including counseling and cognitive behavioral therapy, may be beneficial in helping couples navigate intimacy challenges associated with infertility.^63^

### Digestive symptoms and their impact on sexual function

Endometriosis frequently affects the gastrointestinal tract, leading to symptoms such as constipation, diarrhea, bloating, and nausea, which significantly impact comfort and confidence during sexual activity.^64^^,^^65^ The decision tree model revealed that women with digestive symptoms combined with worsening pain over time exhibited a high probability of sexual dysfunction.

The presence of rectovaginal or bowel-infiltrating endometriosis may lead to pain during defecation (dyschezia), bloating, and increased pelvic pressure, further worsening sexual function.^66^ Additionally, gut dysbiosis and increased inflammation have been implicated in endometriosis pathophysiology, suggesting that gut-targeted therapies, including dietary modifications and probiotics, may play a role in symptom management.^67^

### Body mass index and sexual dysfunction

Body mass index emerged as a significant factor influencing sexual function, with obesity and the combined category of underweight/normal weight (BMI < 25 kg/m^2^) associated with poorer FSFI scores. However, underweight participants were few and were grouped with normal-weight women for analytical purposes. Therefore, no conclusion can be drawn regarding differences between underweight and normal-weight participants specifically.^68^ Future studies with larger samples of underweight women are needed to explore whether distinct associations exist between low-BMI phenotypes and sexual function in endometriosis. Women with obesity and infertility had a high probability of sexual dysfunction, likely due to systemic inflammation, altered hormonal metabolism, and increased pain perception.^69^ Conversely, normal weight was associated with poor sexual function in the absence of infertility, possibly due to hormonal imbalances, reduced estrogen levels, and decreased sexual desire. These findings highlight the need for individualized lifestyle interventions, including weight management strategies, to optimize sexual health outcomes.

### Treatment history and its dual impact on sexual function

Treatment history emerged as an important component of several decision tree pathways. Women who did not receive treatment for endometriosis were more likely to experience sexual dysfunction, suggesting that inadequate symptom control may contribute to persistent sexual impairment. Conversely, some women who had undergone surgical treatment, particularly those over 40 years of age, also exhibited high probabilities of sexual dysfunction. This may be explained by postsurgical complications, persistent pain, adhesions, and decreased ovarian function after surgical treatment of endometriosis.^70^^,^^71^ Surgical interventions, while effective in symptom relief, should be carefully considered in the context of long-term sexual function outcomes.

Importantly, in the CART model, surgical history appeared downstream of symptom-related variables such as pain severity, digestive symptoms, and pain progression, suggesting that surgery is selected by the algorithm after clinical severity markers rather than acting as a primary determinant of sexual outcomes. Interpretation of surgical treatment within the decision tree framework requires caution.^72^^,^^73^ Although surgery is clinically recognized as an effective option to reduce pain and improve quality of life and sexual function in selected patients with endometriosis,^1^^,^^4^^,^^74^ CART models do not estimate causal effects and do not assign intrinsic importance to individual variables in isolation, but rather identify multivariable profiles associated with homogeneous outcomes.^72^^,^^75^^,^^76^

Therefore, the observation that both women who underwent surgery and women who did not undergo surgery appear in terminal nodes with a 100% probability of sexual dysfunction likely reflects different clinical trajectories rather than contradictory effects of surgery itself. Surgical history in this model may serve as a proxy for disease severity, symptom burden, and prior treatment exposure, consistent with the well-described phenomenon of confounding by indication in observational research.^77–79^

Moreover, the cross-sectional design and data-driven modeling strategy do not allow temporal or causal inference regarding treatment effects.^80^ Future studies using longitudinal designs or stratified modeling approaches according to surgical status may better disentangle the independent role of surgery from disease severity and symptom progression.

### Sociodemographic conditions and sexual dysfunction

Being in a couple was generally associated with better sexual function,^81^ possibly due to partner support, emotional security, and shared coping strategies. However, women who were not in a couple and experienced worsening pain over time had a high probability of sexual dysfunction, highlighting the psychosocial vulnerability of single women with endometriosis.^16^ Higher education appeared to be a protective factor, as women with moderate to high education levels had a lower probability of sexual dysfunction. This may be attributed to better health literacy, access to medical care, and proactive disease management strategies.^82^ Smoking was associated with higher rates of sexual dysfunction, potentially due to vascular impairment, increased inflammation, and worsened pelvic pain symptoms.^83^ These findings reinforce the importance of smoking cessation programs in endometriosis care.

### Limitations

This study presents several limitations. Firstly, the sample is not fully representative of French women with endometriosis. The questionnaire was intentionally designed to be simple and accessible, which may have limited our ability to assess certain lifestyle habits in detail. Ethical constraints under French regulations also restricted us from collecting specific personal information, such as participants’ locations. This limitation made direct comparisons with other geographically detailed studies more challenging. These limitations may impact the generalizability of our findings. No confirmatory medical examinations, such as laparoscopic surgery or imaging studies, were required to verify the diagnosis. This introduces the possibility that only women with more severe symptoms or those who had previously received a formal medical diagnosis participated in the study, potentially leading to a selection bias. Additionally, as an Internet-based study, there is a potential for selection bias. Although obesity subclasses (classes I-III) may be clinically relevant, the small number of participants with class III obesity prevented reliable stratified modeling in the decision tree framework. Consequently, obesity severity could not be explored in detail. Given the cross-sectional design, causality between variables cannot be established. In addition, the cross-sectional CART framework does not allow causal interpretation of treatment effects, and the role of surgery may be confounded by disease severity and indication for intervention.

## Conclusion

This study applied a decision tree model to identify key clinical, psychological, and sociodemographic determinants of sexual dysfunction in women with endometriosis. Our findings highlight the complex and multifaceted nature of sexual health impairments in this population, emphasizing the interplay of pain severity, menstrual disorders, infertility, BMI, digestive symptoms, and treatment history. The model successfully categorized women into high- and low-risk groups, demonstrating strong predictive accuracy and providing clinically relevant insights for individualized patient management.

Chronic pelvic pain, particularly dyspareunia, worsening pain over time, and pain during urination or bowel movements emerged as major contributors to poor sexual function, reinforcing the need for comprehensive pain management strategies. Additionally, heavy menstrual bleeding, hormonal imbalances, and infertility-related distress were significant factors negatively impacting sexual well-being. The influence of lifestyle factors such as BMI, smoking, and relationship status further underscores the importance of a multidimensional approach to care. The decision tree methodology proved valuable in uncovering nonlinear relationships and hidden patterns that conventional statistical models might overlook. This underscores its potential as a decision-support tool for healthcare providers, allowing for personalized interventions based on patient-specific risk profiles. Integrating AI-driven predictive models into clinical practice could enhance early identification of vulnerable patients, optimize treatment selection, and improve overall sexual health outcomes in women with endometriosis.

Future research should focus on external validation of this model in diverse populations, exploring its application in longitudinal studies and digital health platforms. The incorporation of machine learning algorithms into gynecological and sexual health assessments represents a promising step toward precision medicine, facilitating more effective and patient-centered care strategies.
